# News and Events of the Week

**Published:** 1910-02-12

**Authors:** 


### News and Events of the Week.

The committee of the "James Watson Lectureship" of the Royal Faculty of Physicians and Surgeons has appointed Dr. Walter K. Hunter lecturer for the ensuing year. Dr. Hunter's lectures will be devoted to the subject of hæmatology.

Three lectures on "Tropical Diseases and their Prevention" will be given at the offices of the Chamber of Commerce by Mr. W. J. Simpson, Professor of Hygiene at King's College, and Lecturer on Hygiene to the London School of Tropical Medicine, on Tuesdays, February 15, 22, and Thursday, March 3, at 4 o'clock. Professor Simpson's lectures will mainly refer to "insect-borne" and "bacterial" diseases in the tropics, and the best methods for their prevention and cure.

The twenty-sixth annual dinner of the Sanitary Inspectors' Association was held on Saturday night last at the Gaiety Restaurant, Strand. Sir James Crichton-Browne (President), who occupied the chair, responding to the toast of "The Sanitary Inspectors' Association," said that fifty years ago the national death-rate was close on 22 per 1,000 living. Only about twenty years ago it stood at 20 per 1,000 living, and for 1908 it was only 14.7.

An important public meeting on "The Promotion of Hygiene and Preservation of Health" is being convened by the Incorporated Institute of Hygiene, and will be held on Monday, February 14, at 4 p.m., at the Royal United Service Institution, Whitehall. Sir William Bennett, K.C.V.O., will preside, and several interesting addresses will be delivered by medical and other authorities. Cards of invitation may be had from the Secretary of the Institute.

Dr. Herbert Williams, medical officer of health of the Port of London, has notified to the Corporation the recent arrival of a steamer off Greenwich conveying, among other cargo, 3,647 frozen carcasses of pigs from Hainan, in China, Many of these had indications that the pigs had suffered from swine fever. Another vessel arrived later on from China with 2,621 similar carcasses. Of these 800 were entire and the remainder were without backbones. There were also on board 51 packages of "pigs' bungs" with similar indications of swine fever. The Local Government Board has upheld the medical officer's view of the matter, requiring the exportation of the carcasses, with the result that all will be re-exported, probably in the form of bacon.

At a recent meeting of the Leicester Guardians a resolution was moved to the effect that no more dead bodies should be sent to the Anatomical Society at Oxford University. The mover stated that in his opinion poor people never benefited by medical science, but were exploited while they were alive and when they were dead. It was the bodies of the rich that should be handed over for anatomical research, for it was the rich who gained by such knowledge. The seconder, a lady, said they all knew the importance of anatomical experiments, but as a Guardian she objected to sending the bodies of those who had been under their care to Oxford. It was pointed out that twenty-one bodies had been sent to the Anatomical Society at Oxford in three years, and that the whole matter has already been most carefully considered. After discussion the resolution was lost by 24 votes to 13.

Dr. C. M. Wenyon and a laboratory assistant have left England on a medical research expedition to Baghdad, under the auspices of the London School of Tropical Medicine.

A movement is on foot to raise some permanent memorial to the late Dr. S. H. Ramsbotham, of Leeds and Harrogate. A committee has been formed to collect subscriptions, and donations to the amount of £250 have already been promised.

At present there is no memorial of the foremost man of science of the thirteenth century, Roger Bacon, who foreshadowed many of the discoveries of modern science, and who is known universally as the inventor of gunpowder. A committee has now been formed to supply this deficiency. Contributions, however small, towards the erection of a memorial, in a suitable place, of this great Englishman will be received by Mr. Oscar Guttmann, 60 Mark Lane, E.C.

The Fulham Finance Committee reported that they had considered the application of Mr. H. Davies (vaccination officer) for an increase of fees. In 1900, when advertising for applicants for the post, it was stated in the advertisement that the estimated remuneration of the office would be about £200 per annum. In 1909 the fees amounted to £190, and the Committee recommended that, subject to the sanction of the Local Government Board, the sum of £10 be paid to Mr. Davies in consideration of the reduction in his fees during the past year. The report was adopted.

The Edith Pechey Phipson Post-Graduate Scholarship, of the value of £40, will be awarded at the end of March, 1910, for the assistance of medical women in any sort of post-graduate study, whether medical, surgical, pathological, or scientific. The scholarship will be awarded to the most deserving applicant, preferably to a woman coming from India, or going to work in India. Applications for the year 1910 will be laid before the Sub-Committee of the Pechey Phipson Memorial Fund, and should be addressed to the Hon. Secretary of the Fund, Mrs. E. M. Slater, 33 Chepstow Villas, London, W.

A Medical Mission Institute was recently opened at Tübingen, says the quarterly paper of the Edinburgh Medical Missions. The Medical Faculty of Tübingen University expressed its interest in the new undertaking by conferring the honorary degree of Doctor of Medicine on Herr Paul Lechler, the donor of the building. The Institute is entirely free of debt, as is also the site on which it stands, and on which a deaconess home and a small hospital for tropical diseases are still to be erected. A sum of over £1,000 is on hand for the general expenses, and funds are being raised for the proposed hospital. It is intended that medical mission students, while studying at the University and taking their degrees there in common with other medical students, may find a home in the Institute with like-minded companions, and receive at the hospital special training, not otherwise obtainable, in the treatment of the tropical diseases they are likely to meet with in their after career. The Institute is now at work under the direction of Dr. Fiebig and Dr. G. Olpp, formerly in China. Eight students have begun the full training, and six missionaries are taking the one-year course.

Last week's issue of *Vanity Fair* contains an admirable sketch portrait, by Elf, of Mr. J. Bland-Sutton, F.R.C.S.

So far as can be ascertained, there has been no appreciable difference in the health statistics of Paris in consequence of the inundation, writes the *Times* Correspondent. There is no sign of any epidemic of zymotic diseases or even of influenza.

On Thursday evening, February 17, Mr. Lionel Tayler, M.R.C.S., L.R.C.P., will read a paper on "Types of Children: their Observation and Nurture." The meeting will commence at 7.30, and the chair will be taken by Dr. Shuttleworth. Light refreshments will be served after the meeting.

The *Standard* has opened its columns to a full discussion of vivisection. The exposition of the case in favour of experiments on animals is in the able hands of Mr. Stephen Paget, while the secretary of the British Union for the Abolition of Vivisection defends the case against vivisection.

Last Monday the Bishop of Ely opened, in the Guildhall, Cambridge, the Tuberculosis Exhibition which at the end of last year was very largely visited in the East End of London. Professor Howard Marsh, Master of Downing College, explained the objects of those who had promoted the exhibition, and during the week a series of lectures has been given by Dr. Niven, M.O.H. for Manchester; Dr. Kerr, Medical Officer, London County Council; Dr. Cobbett, Lecturer in Pathology in the University of Cambridge; and Miss Cummins, Almoner at St. Thomas's Hospital, London.

The death of Mr. William Herbert Hillyer, ship's doctor on the P. and O. *Nore*, who was found in his cabin shot through the head, on February 4, was the subject of an inquest at Stratford last Monday. On his desk was a telegram, which read, "Have failed. My brain has gone. Please break it to Memsahib." Evidence was given that he had been in poor health for the last four years, and Dr. R. W. Doyne said that he had attended Mr. Hillyer, who complained of failing sight. He had had some unsuccessful litigation. While he was making his preparations for a voyage in the *Nore*, however, he lost all power of thinking for himself. The jury returned a verdict of "Suicide while temporarily insane."

An International Exhibition of Hygiene is being organised under the auspices of the Argentine Government by an influential committee of physicians and surgeons resident in Buenos Aires, the President being Dr. Canton, and the Secretary Dr. Luis Agote. In connection with the exhibition a congress of hygiene will be held, and it is intended to organise displays of physical exercises and sporting competitions in the Stadium in the centre of the grounds. The management of the British section is in the hands of a small committee of six members, of which his Majesty's Minister to the Argentine Republic is Honorary Chairman and Dr. Mulcahy Chairman. The exhibition will open on May 25, 1910, and will remain open for a period of not longer than six months. The grounds and buildings will be open daily from 10 A.M. to 6 P.M., and the exhibition will, if the committee considers it desirable, be open on such occasional evenings as may be convenient. A plan of the grounds can be seen on application to the British Commissioner for the Buenos Aires Exhibitions, Queen Anne's Chambers, Westminster, London, S.W.

The Carnegie Hero Fund Trustees have agreed to allow £35 per annum, with a supplement of £15 for the first year in consideration of special circumstances, to the widow and family of Dr. John Herbert Wells, of the Therapeutic Inoculation Department of St. Mary's Hospital, London. It will be remembered that in February 1908 Dr. Wells undertook pioneer investigation into the treatment of glanders, and was able to save the life of a patient who suffered from it. In the course of laboratory work for the case the doctor contracted the infection, and he died in October last year after a painful illness.

Dr. M'Dougall, in reporting on the condition of the children at the Manchester David Lewis Epileptic Colony School, states that the policy of sending children, even at public expense, to a special residential school on the first appearance of epileptic symptoms is a policy of true economy. Quite a number of the children sent from Manchester to this colony have become quite free from fits, and are now earning their living. It seems certain that but for their removal to the special school they would have developed chronic epilepsy, and have become a life-long burden to the rates. A second school would be opened shortly at Soss Moss, near Alderley Edge.

At Clerkenwell last week, Charles Banks, of Cromer Street, Gray's Inn Road, was summoned under the Medical Act for falsely pretending to be a doctor and for giving a certificate of death. Mr. Bodkin, for the Medical Defence Union, said the register of deaths had been rendered false by certain entries. The defendant lived in the upper part of a house, without name-plate or other indication; but he had acquired the name of doctor in the neighbourhood. Mr. Bodkin read a certificate of death said to have been given by "Dr. Banks." The registrar, he said, had some doubt about the certificate as it was not the ordinary form. The certificate was not valid, and the entry had to be corrected by statutory declaration. Mr. Tyrrell, on behalf of the Medical Defence Union, visited the defendant, who said he was consumptive and made out a certificate of ill-health. Mr. Tyrrell was in excellent health, and with such certificates benefit and other societies could be defrauded. The defendant had really no qualifications. Evidence was given to show that the defendant was not registered as a medical man, but had attended a Mrs. Davison for bronchitis. Her husband thought the defendant was an ordinary medical man. Mrs. Davison died, and a certificate signed by Banks was received by the registrar. Thomas William Tyrrell, a solicitor's clerk, said he called on the defendant, who suggested a walk, which ended at the first public-house. There the defendant felt his pulse and said the witness was very weak and was suffering from consumption. The defendant proposed a convalescent home, and said that if he were to enumerate all the complaints from which the witness suffered he would require a very large piece of paper. They then went to a drug stores, where the defendant went into a dispensing room and wrote a certificate stating that the witness would be greatly benefited by a change of air and surroundings. The defendant charged him 5s., and the witness had to pay 1s. 9d. to have the prescription made up. The defendant suggested that they should have a drink, and they again went to the public-house, where the defendant declared that he had a very large practice. The defendant said he was in a very unfortunate position; he would rather say nothing about it. Mr. Bros imposed penalties amounting to £25, with the alternative of three months' imprisonment.

